# COVID-19 Pandemic Awareness, Attitudes, and Practices Among the Pakistani General Public

**DOI:** 10.3389/fpubh.2021.588537

**Published:** 2021-06-09

**Authors:** Rehana Rehman, Shireen Jawed, Rabiya Ali, Khola Noreen, Mukhtiar Baig, Javaria Baig

**Affiliations:** ^1^Department of Biological and Biomedical Sciences, Aga Khan University, Karachi, Pakistan; ^2^Department of Physiology, Aziz Fatima Medical and Dental College, Faisalabad, Pakistan; ^3^Department of Physiology, Karachi Institute of Medical Sciences, Karachi, Pakistan; ^4^Department of Community Medicine, Rawalpindi Medical University, Rawalpindi, Pakistan; ^5^Department of Biochemistry, King Abdulaziz University, Jeddah, Saudi Arabia; ^6^Liaquat College of Medicine and Dentistry, Karachi, Pakistan

**Keywords:** COVID-19, knowledge, attitude, practices, Pakistan

## Abstract

**Background:** Outbreak of COVID-19, in many countries, has imposed a lockdown on their residents. The usefulness of extenuative actions is extremely reliant on society's knowledge, attitudes, and practices (KAP) toward pandemic control.

**Objective:** This study aimed to explore the awareness, attitudes, and practices of the general Pakistani population to COVID-19.

**Methods:** From June 13, 2020, until June 30, 2020, a cross-sectional online KAP survey was conducted among the Pakistani public. For data collection, a validated self-administered questionnaire was used. The survey instrument consisted of six demographic characteristics, 14 items on knowledge, four on attitudes, and six items on practices, modified from a previously published questionnaire on COVID-19.

**Results:** The present study included 2,307 participants, 58.3% males and 41.7% of females. The majority (86.7%) sought information from social media (SM) and television, 95% had good practices, 89.9% had positive attitudes, and two-thirds (67.4%) of the respondents had adequate knowledge. The students and people from younger age groups had more positive attitudes compared with others. Highly educated w with other groups (*p* < 0.001). In logistic regression analysis, the odds ratio indicated that the private job was negatively associated, and high monthly income was positively associated with adequate knowledge (OR = 0.595). Old age was the predictor of negative attitude, and high school degrees and master's degrees were associated with good practice scores.

**Conclusion:** The Pakistani general population has an overall positive attitude and proactive practices against COVID-19, but their knowledge is inadequate. The most important source of information was SM, followed by television. These are playing a crucial role in educating the Pakistani public.

## Introduction

The modern name for the coronavirus is severe acute respiratory syndrome coronavirus 2 (SARS-CoV-2) (1). The coronavirus disease (COVID-19) has been recognized as the root cause of this epidemic of respiratory problems in Wuhan, Hubei Province, China, beginning in December 2019 (2). The current COVID-19 epidemic has disseminated very rapidly by January 31, 2020, and propagated to 19 countries with 11,791 diagnosed cases, including 213 deaths due to the virus (2), and by February 15, 2020, the virus had outstretched in almost 26 countries leading to 51,857 diagnosed cases and 1,669 deaths, with nearly all deaths occurring in China (3). This disease spread worldwide in just a few months and became a global pandemic (4). In reaction to these severe concomitances, the World Health Organization (WHO) declared it a pandemic on January 30, 2020 and ordered combined efforts of all countries to prevent the rapid spread of COVID-19 (3). WHO confirmed that several cases with pneumonia of unknown origin were associated with a local Huanan South China seafood market in Wuhan in December 2019. Still, no specific animal association was declared (2, 5). In China, 19% of cases with COVID-19 developed the severe stage of acute respiratory distress syndrome and coagulation disorders (3). Observed clinical data have shown that the approximate case fatality rate of COVID-19 is 2.4% in China, lower than those of SARS (10%), MERS (35%), and H7N9 (39%) (3).

On February 26, 2020, the first positive case of coronavirus disease (COVID-19) was identified in Pakistan. To counter the spread effectively, Pakistan's government shut down all educational institutions, religious schools, mosques, and leisure spots in the 2nd week of March 2020 (6). All meetings and services have been postponed, marriage halls closed, and all sporting events have been canceled. In addition, a full lockdown was enforced in the country on March 23, 2020 (6). Yet in Pakistan, 80% of patients with COVID-19 have mild symptoms and recover spontaneously, and patients with co-morbidities are more likely to have a serious infection (7). The most basic prerequisite at this point is to learn more about coronaviruses in order to control this pandemic (7). In a struggle to alleviate the COVID-19 pandemic, many countries have enforced a hard lockdown for public movement control and social distancing (8). The usefulness of these alleviation methods is highly based on the society's collaboration and acquiescence (8). However, a study with Asian healthcare workers and medical students explored that they had inadequate information about COVID-19 but had a positive attitude for preventing and controlling COVID-19 (9, 10). Peoples' KAP toward the pandemic has an integral function in revealing the society's preparedness to receive behavioral change measures from the health ministry (8). Knowledge and attitude are related to the degree of fright, panic, and anxiety, which could further complicate actions to control the pandemic (8). Our study aimed to determine the KAP of the Pakistani people toward COVID-19. Our study results could help policymakers devise a sound policy to control this and future health crises.

## Methodology

### Study Design and Setting

A cross-sectional survey was conducted from June 13 to June 30, 2020, after seeking ethical approval (Reference number. 88/1REF/RMU/2020). The data collection procedure complied with the institutional and national ethical guidelines and in accordance with the Helsinki declaration. Anonymity and confidentiality of data were maintained. Non-probability convenience sampling technique was used for this study. Data were collected from the general population from different provinces of Pakistan. The representative sample to attain the required sample size and statistical power size was obtained by Raosoft sample size calculator, which was 664 at a confidence level of 99% with a margin of error of 5%, response distribution 50%, and population size 212.21 million. However, the estimated sample size was inflated to access many participants and gather the maximum possible data to enhance the study's validity and generalizability.

### Data Collection Procedure

Since the general public's routine social activities are suspended, and they are required to observe social distancing and restricted movement due to lockdown, home isolation, and quarantine, in the current scenario, data were collected through an online self-reported data collection tool. The questionnaire was shared through social media and networking sites to obtain the maximum possible data.

Participants were provided with detailed information about the questionnaire, objectives, and purpose of the study before filling the proforma. Anonymity and confidentiality of data were maintained by assuring that information provided will be kept highly confidential and will not be divulged anywhere and solely used for research purposes. If they do not feel comfortable providing any information, they were given the option of skipping that question and were allowed to withdraw at any stage during the study.

All participants were required to give consent for their voluntary participation at the beginning of the study by selecting the yes option after the brief description of the study's purpose and objectives. The participants who gave permission for voluntary participation were allowed to proceed further to complete the questionnaire and submit it online.

### Data Collection Instrument

A self-administered questionnaire was developed after an extensive literature search of already published studies (3, 11) and according to the World Health Organization guidelines (12). An initial draft was validated by two senior faculty members of different departments. After expert review, the draft was revised, and necessary amendments were made to finalize the tool as suggested by the panel. A pilot study was conducted on 35 students for its understanding and checking its reliability. Its Cronbach's alpha was found to be 0.79.

The questionnaire consisted of four parts. Questions related to KAP had three options, true/false/not sure or yes/no/sometimes type; one score was given for true and zero for false and not sure. An individual score of 1–10 was taken as poor, while the score in the range of 11–14 was counted as good. The second part was regarding the assessment of knowledge about COVID-19 (K1–K14), and the third part, related to attitude, had four questions (A1–A4), and the scores were awarded +1 for true and −1 for false and not sure. So the total score ranged from −4 to +4. The plus scores were taken as positive attitudes, while negative scoring indicated negative attitudes. There were six questions related to practice (P1–P6); two points were awarded for yes, one for sometimes, and zero for no, and the score >6 was taken as adequate, and < =6 was taken as inadequate practices (11).

### Statistical Analysis

Statistical analysis was performed by Statistical Package for the Social Sciences version 21 **(**SPSS 21). Categorical variables like gender and responses to each question were expressed as frequencies and percentages. Each response concerning KAP and misconception scores was compared according to the different study variables like age, marital status, education level, monthly income, and job status of the study participants, using the chi-square test (X^2^). Binary logistic regression analysis was used to explore the association among the KAP scores. Results of logistic regression analysis were presented as OR. We took the *p*-value ≤ 0.05 and *p*-value ≤ 0.001 as significant and highly significant, respectively.

## Results

After excluding a few incomplete responses, 2,307 participants, [1,346(58.3%) males and 961(41.7%)], were included in the sample. The general characteristics of the participants are shown in Table 1 (Supplementary Material). Study participants' KAP regarding the COVID-19 pandemic is described in Table 1.

**Table 1:** Study participants' knowledge, attitudes, and practices regarding COVID-19 pandemic.

**Questions**	**Statements**	**Frequency**	**Percentage**
	**Knowledge**	**Correct answer**	
K1	COVID-19 infection is caused by SARS-CoV-2.	1,368	59.3%
K2	COVID-19 infection is spread via respiratory droplets of the infected person.	2,156	93.5%
K3	All community members are equally at risk for COVID-19.	1,645	71.3%
K4	The best way of preventing spread of COVID-19 is social distancing.	2,191	95%
K5	The virus is human-made and deliberately released.	867	37.6%
K6	Any type of group activity may spread this infection.	2,018	87.5%
K7	A symptomless COVID-19 patient (during incubation period) can't transmit infection.	1,707	74%
K8	The risk of getting infected when traveling by plane is higher.	1,197	51.9%
K9	This virus infection can be avoided by frequent hand washings by soap.	2,079	94.5%
K10	Advising quarantine to passengers coming from infected areas is a good practice to avoid spread of infection.	2,237	97%
K11	Lockdown all over the country will control the spread of this virus.	1,870	81.1%
K12	Closing teaching institutions and shopping malls are effective ways of social distancing.	2,044	88.6%
K13	The most common cause of spread of this infection in any country is traveler from infected area.	1,830	79.3%
K14	Isolation period for infected people and those exposed to infection is 14 days.	1,864	80.8%
	**Attitude questions**	True answer	
A1	I am sure that COVID-19 infection will be overcome soon.	1,013	43.9%
A2	We can overcome this problem by taking precautionary steps.	2,115	91.7%
A3	I understand that this infection is highly contagious.	2,144	92.9%
A4	It is my social responsibility to take safety measures in controlling spread of this infection.	2,271	98.4%
	**Practice questions**	Yes	
P1	I am avoiding meeting my friends and relatives.	1,802	78.1%
P2	I am avoiding visiting crowded place.	2,104	91.2%
P3	I am avoiding using ATM machine.	1,541	66.8%
P4	I prefer to walk by stairs than using lift.	1,757	76.2%
P5	I am using face mask outside the home.	1,996	86.5%
P6	I am using soap frequently for handwashing.	2,126	92.2%

Most (71.3%) participants realized that all community members are equally vulnerable to COVID-19, and 74% of the participants believed that COVID-19 patients without symptoms or during the incubation period could not transmit the infection to other individuals. Four out of five respondents believed that travelers from infected areas are the common reason for spreading this infection in any country, and 97% of the population claimed that the spread of infection could be avoided by urging travelers arriving from infected areas to observe quarantine (Table 1).

The participants with a professional degree and Ph.D. had better knowledge compared with other groups. The participants with higher incomes also responded to have better knowledge. Students and government job respondents had adequate knowledge compared with other groups. The majority of the participants (86.7%) sought information regarding COVID-19 from SM, followed by TV (80.6%), government services (50.8%), newspapers (46%), and others (27.8%). Concerning study subjects' practices, we also noticed that 91.2% avoided visiting crowded places. Most of the participants (86.5%) were used to wearing face masks outside, and it was believed to be an effective prevention technique for COVID-19. The study participants (92.2%) frequently washed their hands, and 76.2% chose to use stairs rather than the lift to avoid exposure to COVID 19 (Table 1).

Surprisingly, 1,440 (62.4%) of the participants believed that “the virus is human-made and deliberately released.” In one quarter of the participants, 600 (26%), thought that “a symptomless COVID-19 patient (during the incubation period) couldn't transmit infection,” and 939 (40.7%) could not recognize “SARS-CoV-2 causes COVID-19 disease.”

On analyzing attitude, we found that most of the population had positive attitudes (92.9%), and 98.4% of the participants knew this infection is extremely contagious, and the taking of protective measures to control the spread of this infection is a social responsibility.

**Figure 1:**
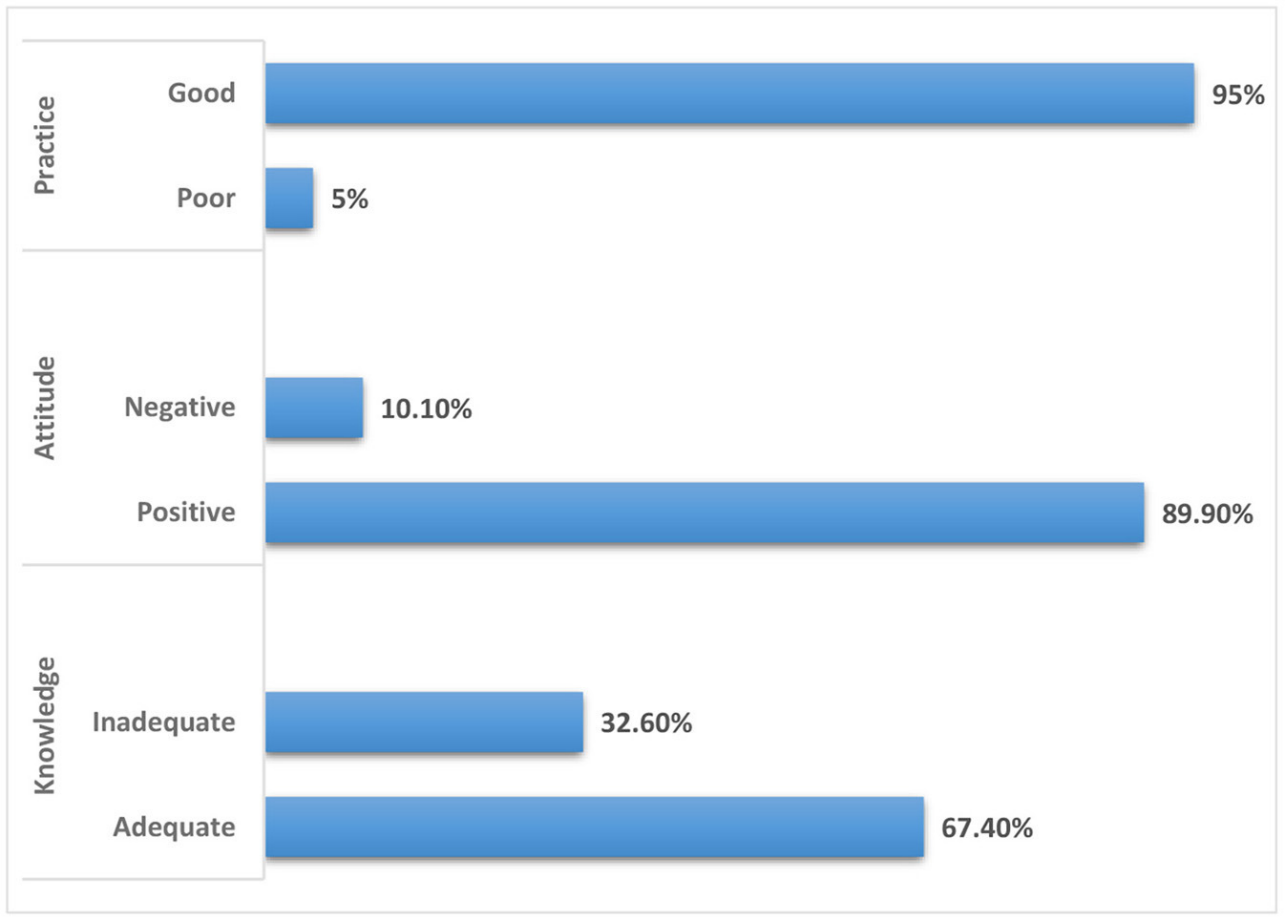
Study participants' knowledge, attitude, and practice categories.

Figure 1 categorizes individuals on the basis of knowledge (adequate/inadequate), attitude (positive/negative), and practices (good/bad). Majority of the participants had good practices [2,192 (95%)], positive attitude [2,073 (89.9%)], and two thirds of the respondents [1,554 (67.4%)] had sufficient information on COVID19 (Figure 1).

Students and people from the younger age groups (18–28, 29–39, and 40–50 years) had more positive attitudes compared with the earning people, and old age (51–60 and >60 years). People not doing any job (students) and housewives' attitudes were more positive than other groups. Married and unmarried people's attitudes were more positive in comparison with divorced subjects. Highly educated people (high school degree, professionals, Ph.D.) demonstrated good practice in contrast with other groups (*p* < 0.001) (Table 2).

**Table 2:** Comparison of knowledge, attitude, practice, and misconception scores according to different variables.

		**Knowledge**		**Attitude**		**Practice**	
		**Inadequate *N* (%)**	**Adequate *N* (%)**	**Negative *N* (%)**	**Positive *N* (%)**	**Poor *N* (%)**	**Good *N* (%)**
Age	18–28	562 (31.7)	1,211 (68.3)	149 (8.4%)	1,624 (91.6%)	94 (5.3)	1,679 (94.7)
	29–39	73 (36.1)	129 (63.9)	22 (10.9%)	180 (89.1%)	7 (3.5)	195 (96.5)
	40–50	46 (32.2)	97 (67.8)	20 (14%)	123 (86%)	6 (4.2)	137 (95.8)
	51–60	47 (37.6)	78 (62.4)	28 (22.4%)	97 (77.6%)	7 (5.6)	118 (94.4)
	>60	24 (37.5)	40 (62.5)	15 (23.4%)	49 (76.6%)	2 (3.1)	62 (96.9)
	*p*-value	0.414		<0.001		0.719	
Education level	Less than high school	8 (40)	12 (60)	3 (15%)	17 (85%)	2 (10)	18 (90)
	High school degree	161 (31.5)	350 (68.5)	47 (9.2%)	464 (90.8%)	12 (2.3)	499 (97.7)
	Bachelor's degree	358 (34.9)	669 (65.1)	112 (10.9%)	916 (89.1%)	78 (7.6)	950 (92.4)
	Master's degree	93 (39.6)	142 (60.4)	28 (11.9%)	207 (88.1%)	15 (6.4)	220 (93.6)
	Professional degree	122 (25.7)	353 (74.3)	38 (8%)	437 (92%)	8 (1.7)	467 (98.3)
	Ph.D	9 (23.7)	29 (76.3)	6 (15.8)	32 (84.2)	1 (2.6)	37 (97.4)
	*p*-value	0.001*		0.288		<0.001*	
Monthly income	<30,000 rupees	64 (39.3)	99 (60.7)	19 (11.7)	144 (88.3)	12 (7.4)	151 (92.6)
	30,000–50,000 rupees	64 (39.3)	99 (60.7)	24 (14.7)	139 (85.3)	1,297.40	151 (92.6)
	50,001–100,000 rupees	53 (27.9)	137 (72.1)	26 (13.7)	164 (86.3)	7 (3.7)	183 (96.3)
	>100,000 rupees	63 (25.5)	184 (74.5)	35 (14.2)	212 (85.8)	9 (3.6)	238 (96.4)
	None	507 (32.9)	1,033 (67.1)	130 (8.4)	1,414 (91.6)	76 (4.9)	1,468 (95.1)
	*p*-value	0.007		0.003		0.243	
Job	Govt job	33 (25.8)	95 (74.2)	20 (15.6%)	108 (84.4%)	4 (3.1)	124 (96.9)
	Private job	155 (42)	214 (58)	49 (15.9%)	311 (84.1%)	20 (5.4)	350 (94.6)
	Business	19 (35.2)	35 (64.5)	10 (17.9%)	46 (82.1%)	5 (8.9)	51 (91.1)
	Housewife	45 (36.3)	79 (63.7)	12 (9.7%)	112 (90.3%)	4 (3.2)	120 (96.8)
	Student	492 (30.4)	1,127 (69.6)	132 (8.1%)	1,488 (91.9%)	81 (5)	1,539 (95)
	*p*-value	<0.001		<0.001		0.443	
Marital status	Married	189 (35)	351 (65)	74 (13.7%)	466 (86.3%)	25 (4.6)	515 (95.4)
	Unmarried	543 (31.5)	1,183 (68.5)	150 (8.7%)	1,576 (91.3%)	89 (5.2)	1,637 (94.8)
	Divorced	2 (15.4)	11 (84.6)	3 (23.1%)	10 (76.9%)	0 (0)	13 (100)
	*p*-value	0.132		0.001		0.629	

In logistic regression analysis, the OR indicated that the participants with high monthly income were the predictors of adequate knowledge. The private jobs were negatively associated with adequate knowledge (OR = 0.595) (Table 3). Age 51–60 years and greater than 60 years were the predictors of negative attitude. High school degrees and master's degrees were associated with good practice scores (Table 3).

**Table 3:** Binary logistic regression analysis showing association of knowledge, attitude, and practice scores.

**Variables**	**Knowledge score**				**Attitude score**				**Practice score**			
	**B**	**SE**	***P*-value**	**OR**	**B**	**SE**	***P*-value**	**OR**	**B**	**SE**	***P*-value**	**OR**
Age			0.736				0.035				0.782	
29–39 years	0.014	0.255	0.956	1.014	−0.508	0.398	0.202	0.602	0.749	0.581	0.197	2.114
40–50 years	0.175	0.291	0.548	1.191	−0.762	0.437	0.081	0.467	0.570	0.642	0.375	1.768
51–60 years	−0.082	0.306	0.790	0.922	−1.308	0.443	0.003	0.270	0.417	0.634	0.511	1.517
>60 years	0.392	0.408	0.336	1.480	−1.085	0.517	0.036	0.338	19.103	5.545	0.997	1.977
Monthly income			0.000				0.903				0.454	
30,000–50,000 rupees	0.032	0.242	0.896	1.032	−0.176	0.347	0.612	0.839	−0.123	0.459	0.789	0.885
50,001–100,000 rupees	0.491	0.245	0.045	1.635	0.032	0.347	0.926	1.033	0.623	0.517	0.228	1.864
>100,000 rupees	0.692	0.249	0.005	1.997	0.139	0.347	0.688	1.149	0.608	0.519	0.241	1.836
None	−0.164	0.191	0.391	0.849	0.044	0.288	0.877	1.045	0.180	0.366	0.623	1.197
Education level			0.077				0.108				0.000	
High school degree	0.135	0.513	0.792	1.145	0.142	0.679	0.834	1.153	1.810	0.846	0.032	6.113
Bachelor's degree	0.087	0.507	0.864	1.091	0.196	0.665	0.768	1.217	0.672	0.798	0.400	1.958
Master's degree	0.156	0.522	0.765	1.169	0.774	0.691	0.263	2.169	0.586	0.842	0.486	1.798
Professional degree	0.483	0.514	0.347	1.621	0.676	0.681	0.321	1.966	2.117	0.865	0.014	8.307
Ph.D	0.388	0.647	0.549	1.474	0.403	0.820	0.623	1.497	0.950	1.316	0.471	2.585
Job			0.000				0.466				0.873	
Private job	−0.519	0.246	0.035	0.595	−0.176	0.320	0.582	0.838	−0.196	0.588	0.739	0.822
Business	−0.174	0.387	0.653	0.840	−0.158	0.468	0.736	0.854	−0.670	0.770	0.384	0.512
Housewife	−0.091	0.324	0.778	0.913	−0.011	0.478	0.981	0.989	−0.648	0.808	0.422	0.523
Student	0.445	0.295	0.131	1.560	0.410	0.410	0.318	1.507	−0.230	0.661	0.728	0.794
Marital status			0.445				0.126				0.879	
Unmarried	−0.089	0.222	0.690	0.915	−0.563	0.342	0.100	0.570	0.245	0.483	0.612	1.27
Divorced	0.890	0.792	0.261	2.435	−1.147	0.726	0.114	0.318	18.537	1.023	0.999	1.123

## Discussion

Public health education is an effective measure to prepare the population to face this disastrous health emergency and take preventive measures to reduce this lethal disease. KAP studies help improve awareness, alleviate panic, encourage positive attitudes, and adopt desired healthy practices (13). The COVID-19 pandemic that has emerged in Wuhan, China, in late 2019, has become a global pandemic, spreading to many countries, including Pakistan (7, 14). First, two confirmed coronavirus cases in Pakistan were reported from Karachi and Islamabad on February 26, 2020 (6, 15). Both cases were recently returned from Iran. According to Pakistan's government, the confirmed cases and death rates until July 4, 2020, were 225,283 and 4,619, respectively, while the active cases were 95,570, and total recoveries in Pakistan were 125,094 (16).

In our study, approximately half of the population was aware that SARS-CoV-2 causes COVID-19 disease. Contrary to this, a previous Pakistani study observed that 94.2% of participants stated the cause of COVID-19 (6). The difference in both studies' results could be that the participants were medical, pharmacy, and allied health sciences staff and students in the previous survey. Therefore, their knowledge about the cause of COVID-19 was better than our study participants (general population). Our findings, however, are justified by the studies conducted in Saudi Arabia and Uganda that documented the vital role of SM and television in educating the public about the prevention of COVID 19 (9, 17). Forty-eight percent of the population was satisfied by government efforts to control the spread of the virus and, more importantly, suggest the availability of treatment of infected patients at healthcare facilities to control the pandemic.

Participants were aware of the mode of transmission of COVID-19 and the majority (80.8%) knew that the incubation period is about 1 to 14 days. Similar knowledge about the viral origin, mode of transmission, and incubation period was acquired in the Chinese population by a KAP study (13). Almost all (95%) study subjects knew that the frequent handwashing by soap and social distancing help prevent COVID-19. The majority (87.5%) of participants believed that any group interaction could transmit this infection. Our findings mentioned above are supported by another Pakistani study conducted in Lahore (6).

We noticed that 92.2% frequently washed their hands, and 76.2% of the participants choose to use stairs rather than the lift to prevent contracting COVID 19. Our results agree with a study conducted in Jordan by Alzoubi et al., documenting that frequent handwashing and mask-wearing are protective measures adopted by the Jordanian population (18). A Chinese study by Peng et al. showed more knowledge, positive attitude, and good health practices among participants to prevent lethal infectious disease (13).

It was also noticed that the highly educated people, having professional and master's degrees, showed good knowledge and healthy practices compared with less educated people. It is conflicting to a survey conducted in Uganda that reported the level of knowledge related to COVID-19 was similar irrespective of their professions or qualifications. They agreed that most of Uganda's population used information from international and governmental media, to have adequate knowledge regardless of their qualification (9). According to a recent Pakistani survey, people with a higher salary, married, and had a higher level of education had better knowledge scores (19).

Young participants had more positive attitudes and preventive practices than the older age group subjects. The current study results indicated that participants older than 50 years of age have negative attitudes and practices toward COVID 19. This is in agreement with Olum et al., who reported that younger subjects (<40 years) were more likely to have better knowledge about COVID-19 than older age subjects (9).

It was encouraging to observe that most of the overall Pakistani population (95%) had good practices and positive attitudes toward the COVID-19 preventive measures despite having low knowledge in the majority of the population. Only two thirds of the study participants had adequate knowledge of COVID-19, reflecting a good understanding of the information available in literature and media. However, in contrast to our results, a previous study conducted at Lahore reported that COVID-19 preventive practices were not adequate in the Lahore population despite having higher knowledge and attitude scores than our studied population (6). In the current study, data were collected from multiple sites in Pakistan, so we get overall good proactive practices than the previous study, which was conducted in a single city. Studies conducted in China and Jordan had reported similar scores concerning attitudes and practices regarding COVID-19 in their studied population, as reported by the current study (13, 18). Contrary to our results study from Uganda, they have reported negative attitudes toward COVID-19, and only 70% of the participants of their study had good practices toward COVID-19 (9). Literature, however, supports that medical students in Pakistan, especially females and senior year scholars, exhibited the anticipated levels of knowledge and were keen to depict attitudes and preventive measures toward COVID-19 (20).

The fatalities of SARS-CoV-2 are increasing day by day, severely damaging the country's economy and affecting people's psychology (21). A recent study about the COVID-19 vaccine trends showed that enormous literature is being published about the COVID-19 vaccine and COVID-19 is the priority of most scientists, academics, and institutes, with a strong interest in care and vaccine production. The safest option is to get vaccinated (22).

The current study identified gaps in particular aspects of knowledge that should be concentrated on in the future. Less knowledge in our majority of the population may be due to the use of less credible information sources, and that needs to be addressed urgently. The current study recommends that the health ministry should arrange awareness and educational campaigns to promote precautionary and preventive measures in COVID-19 to reduce this health challenge in Pakistan.

To halt the pandemic, the government must launch movements to curb its deleterious effects. There is a dire need to implement continuous monitoring and awareness campaigns at community level to sensitize the general public regarding precautionary measures like wearing masks, frequent hand washing, and social distancing. Mass media campaigns, awareness lectures at educational institutions, and health promotion programs to promote healthy recommendations in rural and urban areas that dispel misinformation and myths are a way forward.

### Limitations

It was an online survey; since it was put out on social media, there are undoubtedly many people in Pakistan who could not see or even had access to it. Unintentionally, we ignored the perception of those people who do not use the Internet or social media. Moreover, the responses depended on honesty and were affected by the ability to recall; therefore, they may be susceptible to bias recall. However, this study took into account a large sample to determine the level of KAP of Pakistani citizens regarding COVID-19.

## Conclusion

The Pakistani general population has overall positive attitudes and proactive practices against the COVID-19 outbreak; however, their knowledge concerning this issue is inadequate. Essential information sources were SM, TV, and radio. It reflects that these platforms play a crucial role in educating the Pakistani population.

## Data Availability Statement

The raw data supporting the conclusions of this article will be made available by the authors, without undue reservation.

## Ethics Statement

The studies involving human participants were reviewed and approved by Research and Ethical Committee, Rawalpindi Medical University & Allied Hospitals, Rawalpindi. The patients/participants provided their written informed consent to participate in this study.

## Author Contributions

Conceptualization and literature search by RR. Write up and proof reading by SJ. Manuscript write up by RA. Data collection by KN and JB. Data analysis and data interpretation by MB. All authors contributed to the article and approved the submitted version.

## Conflict of Interest

The authors declare that the research was conducted in the absence of any commercial or financial relationships that could be construed as a potential conflict of interest.
