# Supplementary figures and images for: COVID-19 Pandemic Awareness, Attitudes, and Practices Among the Pakistani General Public
Source: Front Public Health. 2021 Jun 9;9:588537. doi: 10.3389/fpubh.2021.588537 (PMC8219954; doi:10.3389/fpubh.2021.588537)

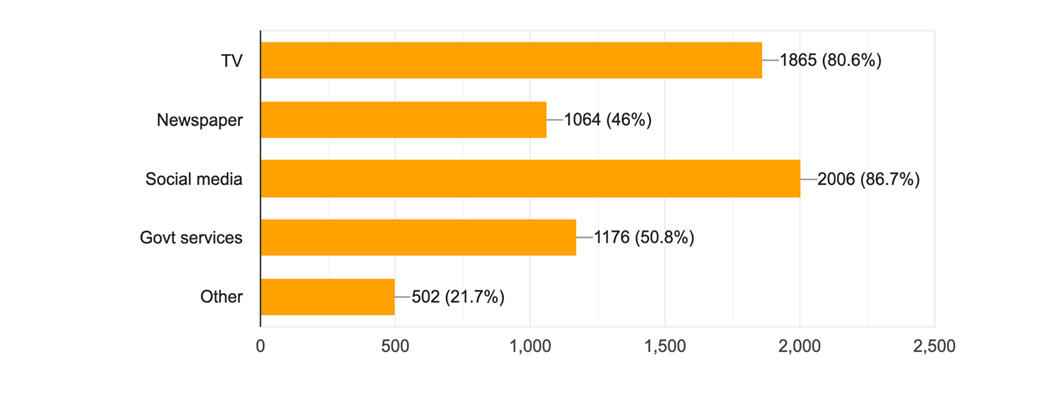


**Fig 1 Sources of information regarding COVID-19**

Supplement: Supplementary file 1 [file Table_2.DOCX]
