# Supplementary material for: COVID-19 Pandemic Awareness, Attitudes, and Practices Among the Pakistani General Public
Source: Front Public Health. 2021 Jun 9;9:588537. doi: 10.3389/fpubh.2021.588537 (PMC8219954; doi:10.3389/fpubh.2021.588537)
Supplement: Supplementary file 2 [file Table_1.docx]

**Table S1. Study participants’ general characteristics (Supplementary data)**

| Variables | | N | % |
| --- | --- | --- | --- |
| Gender | Male | 1346 | 58.3 |
|  | Female | 961 | 41.7 |
| Age | 18-28 | 1773 | 76.9 |
|  | 29-39 | 202 | 8.8 |
|  | 40-50 | 143 | 6.2 |
|  | 51-60 | 125 | 5.4 |
|  | >60 | 64 | 2.8 |
| Education  level | Less than high school | 20 | .9 |
|  | High school degree | 511 | 22.1 |
|  | Bachelor’s degree | 1027 | 44.5 |
|  | Master degree | 235 | 10.2 |
|  | Professional degree | 475 | 20.6 |
|  | Ph.D | 38 | 1.6 |
| Monthly income | <30000 rupees | 163 | 7.1 |
|  | 30000-50000 rupees | 163 | 7.1 |
|  | 50001-100000 rupees | 190 | 8.2 |
|  | >100000 rupees | 247 | 10.7 |
|  | None | 1540 | 66.8 |
| Job | Govt job | 128 | 5.5 |
|  | Private job | 369 | 16 |
|  | Business | 54 | 2.3 |
|  | Housewife | 124 | 5.4 |
|  | Student | 1619 | 70.2 |
| Marital Status | Married | 540 | 23.4 |
|  | Unmarried | 1726 | 74.8 |
|  | Divorced | 13 | .6 |
